# Lack of association between the protein tyrosine phosphatase non-receptor type 22 R263Q and R620W functional genetic variants and endogenous non-anterior uveitis

**Published:** 2013-03-20

**Authors:** María Carmen Cénit, Ana Márquez, Miguel Cordero-Coma, Alejandro Fonollosa, Victor Llorenç, Joseba Artaraz, David Díaz Valle, Ricardo Blanco, Joaquín Cañal, David Salom, José Luis García Serrano, Enrique de Ramón, María José del Rio, Marina Begoña Gorroño-Echebarría, José Manuel Martín-Villa, Blanca Molins, Norberto Ortego-Centeno, Javier Martín

**Affiliations:** 1 Instituto de Parasitología y Biomedicina López-Neyra, IPBLN, CSIC, Granada, Spain; 2 Ophthalmology Department, Hospital de León, León, Spain; 3 Ophthalmology Department, Hospital de Cruces, Bilbao, Spain; 4 Ophthalmology Department, Hospital Clínic, Barcelona, Spain; 5 Ophthalmology Department, Hospital Clínico San Carlos, Madrid, Spain; 6 Rheumatology Department, Hospital Marqués de Valdecilla, IFIMAV, Santander, Spain; 7 Ophthalmology Department, Hospital Marqués de Valdecilla, IFIMAV, Santander, Spain; 8 Ophthalmology Department, Hospital Universitario La Fe, Valencia, Spain; 9 Ophthalmology Department, Hospital Clínico San Cecilio, Granada, Spain; 10 Internal Medicine Department, Hospital Carlos Haya, Málaga, Spain; 11 Ophthalmology Department, Hospital Carlos Haya, Málaga, Spain; 12 Ophthalmology Department, Hospital Universitario Principe de Asturias, Alcalá de Henares, Spain; 13 Immunology Department, Facultad de Medicina, Universidad Complutense de Madrid, Spain; 14 Instituto de Investigaciones Biomédicas, IDIBAPS, Hospital Clinic, Barcelona, Spain; 15 Internal Medicine Department, Hospital Clínico San Cecilio, Granada, Spain

## Abstract

**Objective:**

Endogenous uveitis is a major cause of visual loss mediated by the immune system. The protein tyrosine phosphatase non-receptor type 22 (*PTPN22*) gene encodes a lymphoid-specific phosphatase that plays a key role in T-cell receptor (TCR) signaling. Two independent functional missense single nucleotide polymorphisms (SNPs) located within the *PTPN22* gene (R263Q and R620W) have been associated with different autoimmune disorders. We aimed to analyze for the first time the influence of these *PTPN22* genetic variants on endogenous non-anterior uveitis susceptibility.

**Methods:**

We performed a case-control study of 217 patients with endogenous non-anterior uveitis and 718 healthy controls from a Spanish population. The *PTPN22* polymorphisms (rs33996649 and rs2476601) were genotyped using TaqMan allelic discrimination assays. The allele, genotype, carriers, and allelic combination frequencies were compared between cases and controls with χ^2^ analysis or Fisher’s exact test.

**Results:**

Our results showed no influence of the studied SNPs in the global susceptibility analysis (rs33996649: allelic *P-*_value_=0.92, odds ratio=0.97, 95% confidence interval=0.54–1.75; rs2476601: allelic *P-*_value_=0.86, odds ratio=1.04, 95% confidence interval=0.68–1.59). Similarly, the allelic combination analysis did not provide additional information.

**Conclusions:**

Our results suggest that the studied polymorphisms of the *PTPN22* gene do not play an important role in the pathophysiology of endogenous non-anterior uveitis.

## Introduction

Uveitis is a phenotypically heterogeneous group made up of different clinical entities that have intraocular inflammation in common. This inflammatory condition mainly affects the uveal tract of the eye, located between the sclera, the conjunctiva, and the anterior chamber on the outside and the retina on the inside [1]. Furthermore, this disorder, which usually affects young adults, is classified depending on the anatomic part of the eye affected as anterior uveitis (AU), intermediate uveitis (IU), posterior uveitis (PU), and panuveitis (PAN) [2], and may be triggered by exogenous or endogenous agents. Uveitis can occur either as an isolated condition or as part of a systemic inflammatory disease [3]. In fact, around one third of patients present this condition in association with various systemic disorders such as psoriatic arthritis, reactive arthritis, and Behcet’s disease, among others. AU is the most common clinical form of the disease and is usually associated with HLA-B27-associated seronegative spondyloarthropathies [3]. However, the etiology of IU, PU, and PAN is frequently idiopathic, and these uveitis subtypes typically carry worse outcomes of the disease. Moreover, whereas AU patients generally respond to topical treatment, patients with non-anterior uveitis require systemic treatment [4]. In any case, uveitis is an important socioeconomic problem accounting for about 10% of legal blindness and is the fourth leading cause of blindness worldwide [5,6].

The etiology of autoimmune uveitis is not completely understood, but genetic [7], environmental [8], and immunological factors [9] are involved in the appearance and progression of this trait highlighting in the latter the role of T lymphocytes. Thus far, different genetic variants located within several genes, including genes positioned in the major histocompatibility complex region and the non-major histocompatibility complex region, have been involved in the uveitis predisposition [10-13]. However, the overall genetic component of uveitis, mainly the non-anterior uveitis genetic component, remains unknown. In addition, to date, few studies have been conducted to identify the non-anterior genetic background.

The pathogenesis of endogenous uveitis is essentially mediated by a T cell–driven cellular immune response as in other autoimmune diseases, such as inflammatory bowel disease and type 1 diabetes, among others. In fact, experimental autoimmune uveitis (EAU) induction is characterized by polarization of early T-helpers toward Th1, a T helper cell, while EAU resistance is associated with polarization toward a Th2 pathway [8]. The protein tyrosine phosphatase non-receptor type 22 (*PTPN22*) gene, located within the chromosome region 1p13, encodes a 110-kDa lymphoid-specific phosphatase (Lyp) [14]. Lyp plays a key role as a potent negative downregulator and critical gatekeeper of T-cell receptor (TCR) signaling [15]. Interestingly, the *PTPN22* gene has emerged as an important genetic risk factor for human autoimmunity by multiple mechanisms, including altered thymic selection, reduced T-helper activity, and decreased number or function of regulatory T cells [16-21]. Previously, the possible influence of the *PTPN22* gene in anterior uveitis, the most common form of uveitis, has been investigated [22], but to our knowledge, no previous studies evaluated the possible implication of the functional variants R263Q and R620W of the *PTPN22* gene specifically in non-anterior uveitis.

Two missense single nucleotide polymorphisms (SNPs) have been associated with autoimmune disorders. The R620W (C1858T, rs2476601) polymorphism in *PTPN22* exon 14 was first associated with type 1 diabetes [18] and subsequently with other autoimmune disorders such as rheumatoid arthritis, systemic lupus erythematosus, and systemic sclerosis [19-21]. This substitution occurs within a protein–protein interaction domain. Studies have yielded conflicting results, and the precise effect of the R620W functional genetic variant is not still fully understood. Nevertheless, a clear regulatory role for Lyp in the immune system has been established, and the R620W polymorphism seems to modify the Lyp function in one way or another and consequently to alter the immune response [23,24]. In addition, the R263Q (G788A; rs33996649) polymorphism located within *PTPN22* exon 10 has also been associated with autoimmunity. This polymorphism is located within the catalytic domain of the enzyme, and data have suggested that the minor allele A, a protective factor associated with systemic lupus erythematosus [25], encodes for a loss-of-function variant of Lyp [25]. Taking into account this evidence, in the current study we evaluated the influence of the *PTPN22* R263Q and R620W polymorphisms in the genetic background of endogenous non-anterior uveitis.

## Methods

Five to ten ml of EDTA-treated peripheral blood samples or saliva was collected of each individual and genomic DNA was isolated from peripheral white blood cells or saliva following standard procedures such as Qiagen and Orangene DNA purification kits. DNA was quantified using NanoDrop spectrophotometer and DNA solutions were stored at -80°C until required. DNA dilutions at 10 ng/ul concentration were stored at -20ºC until use for PCR reaction. The SNPs were genotyped using a predesigned TaqMan allelic discrimination assay for the rs2476601 polymorphism (IDs: C__16021387_20) and a custom TaqMan allelic discrimination assay for the rs33996649 genetic variant in a 7900HT Real-Time PCR System from Applied Biosystems (Foster City, CA).

217 uveitis samples were recruited from different hospitals from Spain: Hospital Clínico San Cecilio, Granada; Hospital Clinic, Barcelona; Hospital Marqués de Valdecilla, Santander; Hospital de Cruces, Bilbao; Hospital Carlos Haya, Málaga; Hospital Clínico San Carlos, Madrid; Hospital de León, León; Hospital Universitario La Fe, Valencia and Hospital Principe de Asturias, Alcalá de Henáres. Healthy controls samples were provided by Banco Nacional de ADN from Salamanca and Biobanco Vasco from Bizkaia. The breakdown mean of uveitis patients was 46.3 years and 52.1% of uveitis patients were females. At the time of recruitment all uveitis patients did not have other associated autoimmune diseases. On the other hand, none of the controls had ocular disease. We genotyped the rs33996649 and rs2476601 polymorphisms in a total of 217 patients with endogenous uveitis, excluding the exclusively anterior uveitis forms and uveitis associated with systemic immune-mediated diseases except Vogt-Koyanagi-Harada syndrome, and 718 ethnically matched healthy controls, all from a Spanish population. Written informed consent and approval of the local ethical committees were obtained. The research followed the tenets of the Declaration of Helsinki.

In addition, to examine whether the selected SNPs might influence the different clinical manifestations of the disease, patients with uveitis were subdivided according to the characteristics included in Table 1. The intraocular inflammation seen in patients included intermediate uveitis (17.5%), posterior uveitis (47.9%), and panuveitis (25.8%).

**Table 1:** Clinical and demographic features of uveitis patients.

General characteristics of uveitis patients	All patients n=217
Female (%)	113 (52.1)
Age (mean ± SD)	46.3±15.48
Intermediate Uveitis (%)	38 (17.5)
Posterior Uveitis (%)	104 (47.9)
Panuveitis (%)	56 (25.8)
Bilateral affection (%)	149 (68.7)
Vitritis (%)	135 (62.2)
Macular edema (%)	89 (41.0)
Retinal vasculitis (%)	83 (38.2)
Choroidal neovascularization (%)	20 (9.2)

We performed a case-control study using PLINK (v1.07). The genotype, allele, and carrier frequencies were compared between the patients, patient subgroups, and controls with the χ^2^ test and/or Fisher’s exact test when necessary (when the expected frequencies were lower than 5). Odds ratios (ORs) and 95% confidence intervals (CIs) were obtained according to Woolf’s method. Odds ratios (ORs) and 95% confidence intervals (CIs) were obtained according to Woolf's method. Woolf's method is based in the weight of each stratum giving the most weight to the stratum that have the smallest variance. P values lower than 0.05 were considered statistically significant. However, the effect of the studied genetic variants was analyzed either in isolation or in combination with the conferred effect for the other polymorphism by allelic combination analysis.

## Results

The two studied polymorphisms conformed to Hardy–Weinberg expectations in the control subgroup, and the control allelic frequencies were similar to those previously reported in the European Caucasian population [21]. The genotyping success rate was higher than 95% for both analyzed SNPs. Table 1 summarizes the main features of the patients with uveitis included in the study population, and the genotypic and allelic frequencies in overall patients with uveitis, patients with uveitis stratified according to the anatomic classification [2], and controls are shown in Table 2.

**Table 2:** Allelic and genotype frequencies of *PTPN22* genetic variants in overall uveitis patients, uveitis patients stratified according to the anatomic classification and healthy controls from Spanish population.

**SNP**	**Subgroup (n)**	**GG**	**GA**	**AA**	**MAF (%)**	**Allelic P -value**	**OR (95% CI)**
rs2476601	Controls (n=721)	628 (87.10)	89 (12.34)	4 (0.55)	110 (6.75)		
	Uveitis (n=215)	186 (86.51)	28 (13.02)	1 (0.47)	30 (6.98)	0.86	1.04 (0.68–1.59)
	IU (n=37)	30 (81.08)	7 (19.92)	0 (0.00)	7 (9.46)	0.36	1.45 (0.65–3.24)
	PU (n=104)	88 (84.62)	16 (15.38)	0 (0.00)	16 (7.69)	0.61	1.16 (0.67–2.00)
	PAN (n=56)	52 (92.86)	3 (5.36)	1 (1.78)	5 (4.46)	0.35	0.65 (0.26–1.63)

**SNP**	**Subgroup (n)**	**CC**	**CT**	**TT**	**MAF (%)**	**Allelic P -value**	**OR (95% CI)**
rs33996649	Controls (n=718)	668 (93.04)	49 (6.82)	1 (0.14)	51 (3.55)		
	Uveitis (n=217)	202 (93.09)	15 (6.91)	0 (0.00)	15 (3.46)	0.92	0.97 (0.54–1.75)
	IU (n=38)	37 (97.37)	1 (2.63)	0 (0.00)	1 (1.31)	0.3	0.36 (0.05–2.66)
	PU (n=104)	95 (91.35)	9 (8.65)	0 (0.00)	9 (4.33)	0.58	1.23 (0.60–2.53)
	PAN (n=56)	52 (92.86)	4 (7.14)	0 (0.00)	4 (3.57)	0.99	1.01 (0.36–2.84)

IU; Intermediate Uveitis, PU ; Posterior Uveitis, PAN; Panuveitis, SNP; Single Nucleotide Polymorphism, MAF ; Minor Allele Frequency, OR ; Odds ratio.

No statistical significant differences were observed in the genotype, allele, and carrier frequencies for the rs33996649 and rs2476601 polymorphisms between the patients with uveitis and the controls (rs33996649; allelic *P*_value_=0.92, OR=0.97, 95% CI=0.54–1.75; rs2476601 allelic *P*_value_=0.86, OR=1.04, 95% CI=0.68–1.59). Moreover, no statistically significant differences were apparent when we compared the different subgroups of patients stratified according to the different general characteristics shown in Table 1 for the analyzed polymorphisms.

We also studied the possible additive effect of the two studied polymorphisms in the global disease with allelic combination analysis in the patients with uveitis and the controls. When we inferred the allelic combinations, we observed only three combinations with a frequency >0.01 in the healthy controls and patients (263C–620A [frequency in healthy controls 0.068], 263C–620G [0.897], and 263T–620G [0.036]), and the comparisons of the different detected allelic combinations between the patients with uveitis and the controls did not show significant results (Table 3).

**Table 3:** *PTPN22* allelic combinations (rs2476601-rs33996649) in uveitis patients and healthy controls from Spanish population.

Allelic combination	Uveitis, n (%)	Controls, n (%)	P value	OR [95% CI]
GC	381 (89.4)	1288 (89.7)	0.88	0.97 (0.67–1.41)
AC	30 (7.1)	97 (6.8)	0.84	1.05 (0.67–1.63)
GT	15 (3.5)	51 (3.6)	0.98	0.99 (0.53–1.84)

## Discussion

Endogenous uveitis is an inflammatory response mediated by the immune system driven by a loss of tolerance against ocular antigens. Although the etiology of uveitis remains unclear, humoral and cellular components of the immune response trigger a cascade of events that ultimately lead to the tissue damage detected in this condition [1].

Different genetic factors are shared among the different autoimmune disorders, suggesting that some pathologies may be influenced by common molecular pathways [26]. Recent data have supported that uveitis seems to share different genetic risk factors with other autoimmune diseases, and consequently some of the pathogenic mechanisms involved [7]. Lyp, encoded by the *PTPN22* gene, is a master regulator of autoimmunity important in the negative control activation and development of T-cells [15,27].

The analyzed functional missense polymorphisms in this study have been associated with different autoimmune conditions [16,18-21], although some studies have demonstrated a lack of association of these variants with other autoimmune diseases, such as multiple sclerosis among others [17,28]. One difference between *PTPN22*-associated and non-associated diseases could be the involvement of the humoral component, which appears to be less prominent in the latter. In fact, all diseases associated with the PTPN22*W620 variant are well characterized by the presence of autoantibodies frequently involved in the onset and progression of the disease [29]. In this study, we found no significant association between these two genetic variants and endogenous non-anterior uveitis. The lack of association between the *PTPN22* gene and this disorder suggests that the *PTPN22*-associated diseases share a common underlying mechanism that may not be important in the uveitis pathogenesis. Therefore, the lack of evidence of an association between the *PTPN22* gene and non-anterior uveitis might suggest the low involvement of the humoral component in non-anterior uveitis although further studies are needed.

This study represents the first attempt to evaluate the possible implication of the functional variants R263Q and R620W of the *PTPN22* gene in the pathophysiology of endogenous non-anterior uveitis in a well-defined cohort. In addition, since the two analyzed polymorphisms are independent and both are separately relevant for the *PTPN22* function, uveitis can be either the consequence of mutations present simultaneously in both genetic positions or, alternatively, in only one. Therefore, the effect of the R263Q variant on uveitis was analyzed in isolation and in combination with the effect of the other analyzed genetic variant (R620W). However, these genetic variants did not seem to modify the uveitis predisposition either in isolation or in allelic combination.

Previously, the possible influence of the *PTPN22* gene in anterior uveitis susceptibility was investigated without evidence of association [22]. Different studies have suggested that *PTPN22* is a genetic risk factor for Behcet’s disease [30,31], a disorder that usually presents ocular damage as a major manifestation, although another study found that this gene does not seem to influence the ocular manifestation of this disease [32]. Therefore, our results agree with previously published results and could indicate that the *PTPN22* gene does not play an important role in the pathophysiology of the different clinical forms of uveitis.

As a limitation, our study presents limited statistical power due to the low incidence of endogenous non-anterior uveitis and the low minor allele frequencies of the analyzed polymorphisms. Therefore, this lack of association of the *PTPN22* polymorphisms with uveitis should be interpreted carefully. In the present study, slight effects may not been uncovered, and therefore, our results do not completely rule out the possibility of an association with non-anterior uveitis. A minor effect of the *PTPN22* gene cannot be discarded, and additional studies are required to draw stronger conclusions about the exact role of the R263Q and R620W polymorphisms in the susceptibility and clinical spectrum of non-anterior uveitis.
